# Luteal blood flow in patients undergoing GnRH agonist long protocol

**DOI:** 10.1186/1757-2215-4-2

**Published:** 2011-01-11

**Authors:** Akihisa Takasaki, Isao Tamura, Fumie Kizuka, Lifa Lee, Ryo Maekawa, Hiromi Asada, Toshiaki Taketani, Hiroshi Tamura, Katsunori Shimamura, Hitoshi Morioka, Norihiro Sugino

**Affiliations:** 1 Department of Obstetrics and Gynecology, Yamaguchi University Graduate School of Medicine, Minamikogushi 1-1-1, Ube, 755-8505, Japan; 2 Department of Obstetrics and Gynecology, Saiseikai Shimonoseki General Hospital, Yasuokacho 8-5-1, Shimonoseki, 751-0823, Japan

## Abstract

**Background:**

Blood flow in the corpus luteum (CL) is closely related to luteal function. It is unclear how luteal blood flow is regulated. Standardized ovarian-stimulation protocol with a gonadotropin-releasing hormone agonist (GnRHa long protocol) causes luteal phase defect because it drastically suppresses serum LH levels. Examining luteal blood flow in the patient undergoing GnRHa long protocol may be useful to know whether luteal blood flow is regulated by LH.

**Methods:**

Twenty-four infertile women undergoing GnRHa long protocol were divided into 3 groups dependent on luteal supports; 9 women were given ethinylestradiol plus norgestrel (Planovar) orally throughout the luteal phase (control group); 8 women were given HCG 2,000 IU on days 2 and 4 day after ovulation induction in addition to Planovar (HCG group); 7 women were given vitamin E (600 mg/day) orally throughout the luteal phase in addition to Planovar (vitamin E group). Blood flow impedance was measured in each CL during the mid-luteal phase by transvaginal color-pulsed-Doppler-ultrasonography and was expressed as a CL-resistance index (CL-RI).

**Results:**

Serum LH levels were remarkably suppressed in all the groups. CL-RI in the control group was more than the cutoff value (0.51), and only 2 out of 9 women had CL-RI values < 0.51. Treatments with HCG or vitamin E significantly improved the CL-RI to less than 0.51. Seven of the 8 women in the HCG group and all of the women in the vitamin E group had CL-RI < 0.51.

**Conclusion:**

Patients undergoing GnRHa long protocol had high luteal blood flow impedance with very low serum LH levels. HCG administration improved luteal blood flow impedance. This suggests that luteal blood flow is regulated by LH.

## Background

During corpus luteum formation after the ovulatory LH surge, active angiogenesis occurs and the corpus luteum becomes one of the most highly vascularized organs in the body [1,2]. Blood flow in the corpus luteum is important for the development of the corpus luteum and maintenance of luteal function [3-5]. Adequate blood flow in the corpus luteum is necessary to provide luteal cells with the large amounts of cholesterol that are needed for progesterone synthesis and to deliver progesterone to the circulation [6].

Luteal phase defect has been implicated as a cause of infertility and spontaneous miscarriage. However, luteal phase defect has a complicated etiology and various causes. We recently reported a close relationship between luteal blood flow and luteal function [4]. Interestingly, luteal blood flow was significantly correlated with serum progesterone concentration during the mid-luteal phase, and luteal blood flow was significantly lower in women with luteal phase defect than in women with normal luteal function, suggesting that low blood flow of the corpus luteum is associated with luteal phase defect. Furthermore, we found that luteal phase defect can be improved by increasing luteal blood flow [5]. Therefore, a decrease in luteal blood flow is one of the causes of luteal phase defect.

However, it is still unclear how the decrease in blood flow is caused in patients with luteal phase defect, and how luteal blood flow is regulated in the ovary during the menstrual cycle. Luteal blood flow was increased by HCG administration during the luteal phase [5,7]. Luteal blood flow was also found to be related with serum HCG levels between 5 and 16 weeks of gestation [8]. These findings suggest that HCG or LH has a role in the regulation of luteal blood flow.

Gonadotropin-releasing hormone agonist (GnRHa) has been used to suppress endogenous gonadotropin secretion in standardized ovarian-stimulation protocol for IVF-ET, so called GnRHa long protocol. It is interesting to note that GnRHa long protocol causes luteal phase defect because of remarkable suppression of serum LH levels. Examining luteal blood flow in the patient undergoing GnRHa long protocol would be useful to know whether luteal blood flow is regulated by LH. Therefore, the present study was undertaken to examine luteal blood flow in the patient undergoing GnRHa long protocol.

## Methods

The project was reviewed and approved by the Institutional Review Board of Yamaguchi University Graduate School of Medicine. Informed consent was obtained from all the patients in this study.

### Ultrasonography

Blood flow in the corpus luteum was measured as reported previously [4] using a computerized ultrasonography with an integrated pulsed Doppler vaginal scanner [Aloka ProSound SSD-3500SV and Aloka UST-984-5 (5.0 MHz) vaginal transducer, Aloka Co. Ltd, Tokyo, Japan]. The high pass filter was set at 100 Hz, and the pulse repetition frequency was 2-12 kHz, for all Doppler spectral analyses. After the endovaginal probe was gently inserted into the vagina, adnexal regions were thoroughly scanned. The ovary was identified, and color signals were used to detect the area with the highest blood flow within the corpus luteum. Blood flow was identified in the peripheral area of the corpus luteum [4]. The pulsed Doppler gate was then placed on that area to obtain flow velocity waveforms. An acceptable angle was less than 60°, and the signal was updated until at least four consecutive flow velocity waveforms of good quality were obtained. Blood flow impedance was estimated by calculating the resistance index (RI), which is defined as the difference between maximal systolic blood flow (S) and minimal diastolic flow (D) divided by the peak systolic flow (S-D/S). Blood flow impedances were examined in the corpus luteum during the mid-luteal phase (6-8 days after ovulation). The day of ovulation was determined by urinary LH, transvaginal ultrasonography and basal body temperature records. The cutoff value of the RI of the corpus luteum (CL-RI) was previously determined by receiver operating characteristic curve (ROC) analysis [5]. A cutoff value of 0.51 provided the best combination with 84.3% sensitivity and 85.6% specificity to discriminate between normal luteal function and luteal phase defect [5]. Thus, when CL-RI was more than 0.51, the patient was diagnosed as having decreased luteal blood flow. Since the interobserver coefficient of variation for Doppler flow measurements in the present study was less than 10%, the Doppler flow measurements were judged to be reproducible.

### Clinical studies

Twenty-four patients were enrolled in this study. The mean age was 36.6 years, with a range of 23-43 years. The patients were non-smokers and free from major medical illness including hypertension; they were excluded if they had myoma, adenomyosis, congenital uterine anomaly, or ovarian tumors or if they used estrogens, progesterone, androgens, or had chronic use of any medication, including nonsteroidal anti-inflammatory agents. The patients received artificial insemination with husband's semen (AIH) under the standardized ovarian-stimulation protocol (GnRHa long protocol), consisting of GnRHa (900 mg/day buserelin acetate, Suprecur; Mochida Pharmaceutical Co. Ltd., Tokyo, Japan) beginning in the mid-luteal phase of the previous cycle, followed by 225 IU follicle-stimulating hormone (FSH, Folyrmon-P; Fuji Pharmaceutical Co. Ltd., Tokyo, Japan) on the third day and days 4 and 5, and thereafter by 150 IU human menopausal gonadotropin (hMG, HMG-F; Fuji Pharmaceutical Co. Ltd., Tokyo, Japan). When follicles reached 18 mm or more in diameter by ultrasonography, 10,000 IU human chorionic gonadotropin (HCG, Gonatropin; Asuka Pharmaceutical Co. Ltd., Tokyo, Japan) was administered for ovulation induction. Since the GnRHa long protocol causes luteal phase defect because of low serum LH levels due to GnRHa-induced gonadotropin suppression, the patients received some treatments as a luteal support. Dependent on luteal supports, the patients were randomly divided into 3 groups; 9 women were given ethinylestradiol (0.05 mg) plus norgestrel (0.5 mg) (Planovar, Weis-Ezai Co Ltd., Tokyo, Japan) orally from the day after ovulation induction throughout the luteal phase (control group); 8 women were given HCG 2,000 IU on days 2 and 4 after ovulation induction in addition to Planovar (HCG group); 7 women were given vitamin E (600 mg/day, 3 times per day; Eisai Co., Ltd., Tokyo, Japan) orally throughout the luteal phase in addition to Planovar (vitamin E group). Planovar was used as a control in this study because it did not affect luteal blood flow in our preliminary study [CL-RI of the treatment group and the no treatment group: 0.515 ± 0.073 v.s. 0.505 ± 0.019 (mean ± SEM, n = 11), not significant]. Vitamin E was used to increase luteal blood flow as we reported previously [5]. CL-RI as blood flow impedances in the corpus luteum and serum concentrations of LH, FSH, and progesterone were measured during the mid-luteal phase (6-8 days after ovulation). For patients with multiple ovulations, CL-RI was examined in each corpus luteum, and the mean was used as a patient mean value.

### Statistical analyses

Statistical analysis was carried out with SPSS for Windows 13.0. Kruskal-Wallis test followed by the Mann-Whitney *U*-test using the Bonferroni correction and chi-squared test were used as appropriate. A value of *P *< 0.05 was considered significant.

## Results

Table 1 shows the patient profile of the treatment groups. The numbers of matured follicles and ovulated follicles and serum progesterone concentrations did not significantly differ among the groups (Table 1). Serum concentrations of LH and FSH were remarkably suppressed in all groups (Table 1).

**Table 1:** Profiles of the treatment groups

	n	age	No. of preovulatory follicles (18 mm or greater)	No. of ovulation	serum concentrations		
					
					LH (mIU/ml)	FSH (mIU/ml)	P4 (ng/ml)
Control	9	36.4 ± 1.7	2.1 ± 0.4	1.9 ± 0.4	0.10 ± 0.03	1.20 ± 0.4	38.9 ± 7.8

HCG	8	37.8 ± 1.7	2.8 ± 0.7	2.6 ± 0.5	0.12 ± 0.01	0.94 ± 0.2	56.2 ± 22.9

Vitamin E	7	35.7 ± 1.4	2.3 ± 0.5	2.6 ± 0.8	0.22 ± 0.04	0.85 ± 0.1	32.3 ± 10.9

Twenty-four patients who underwent AIH under the standardized ovarian-stimulation protocol with GnRHa were recruited in this study. The numbers of follicles (18 mm or greater) were measured at the day of HCG injection for ovulation induction. The numbers of ovulated follicles were estimated 2 days after HCG injection. Dependent on luteal supports, the patients were divided into 3 groups; 9 women were given ethinylestradiol (0.05 mg) plus norgestrel (0.5 mg) (Planovar) orally throughout the luteal phase; 8 women were given hCG 2,000 IU on days 2 and 4 after ovulation induction in addition to Planovar (HCG group); 7 women were given vitamin E (600 mg/day, 3 times per day) orally throughout the luteal phase in addition to Planovar (vitamin E group). Planovar was used as a control in this study because it does not affect luteal blood flow. Vitamin E was used to increase luteal blood flow. Serum concentrations of LH, FSH, and progesterone were examined during the mid-luteal phase (6-8 days after ovulation). Values are mean ± SEM. There were no significant differences in any parameters between the three treatment groups.

**Figure 1:**
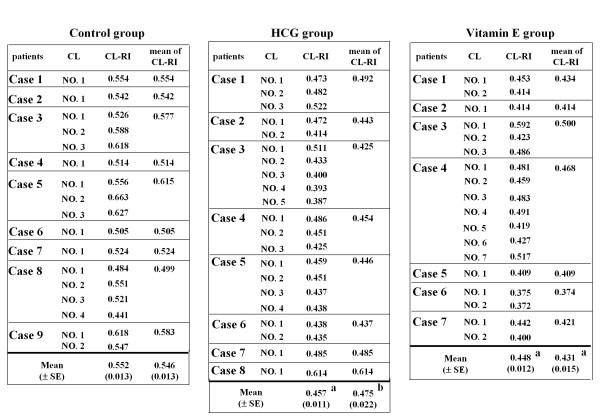
**Corpus luteum-resistance index (CL-RI) of each corpus luteum of each patient in the treatment groups**. Twenty-four patients who underwent AIH under the standardized ovarian-stimulation protocol with GnRHa were recruited in this study. Dependent on luteal supports, the patients were divided to 3 groups; 9 women were given ethinylestradiol plus norgestrel (Planovar) orally throughout the luteal phase; 8 women were given HCG 2,000 IU on days 2 and 4 after ovulation induction in addition to Planovar (HCG group); 7 women were given vitamin E (600 mg/day, 3 times per day) orally throughout the luteal phase in addition to Planovar (vitamin E group). Planovar was used as a control in this study because it does not affect luteal blood flow. Vitamin E was used to increase luteal blood flow. CL-RI was examined during the mid-luteal phase (6-8 days after ovulation). In case of patients with multiple ovulations, CL-RI was examined in each corpus luteum, and the mean was used as a patient mean value. a; p < 0.01 and b; p < 0.05 v.s. control group (Kruskal-Wallis test followed by the Mann-Whitney *U*-test using the Bonferroni correction).

The mean CL-RI of the control group (0.564 ± 0.013) was more than the cutoff value (0.51); only 2 of the 9 patients in this group had CL-RI < 0.51 (Table 2 and Figure 1). Treatments with HCG or vitamin E significantly improved the CL-RI to less than 0.51; only 1 of the 8 patients in the HCG group and none of the 7 patients in the vitamin E group had CL-RI > 0.51 (Table 2 and Figure 1).

**Table 2:** CL-RI of the treatment groups

	No. of patients	CL-RI	No. of patients with CL-RI < 0.51
Control	9	0.546 ± 0.013	2/9

HCG	8	0.475 ± 0.022^b^	7/8^c^

Vitamin E	7	0.431 ± 0.015^a^	7/7^c^

The table summarizes the data in Figure 1. Corpus luteum-resistance index (CL-RI) was measured during the mid-luteal phase in the control group, HCG group, and vitamin E group. The value shows the mean ± SEM from the patient mean value. In this study, when CL-RI was more than 0.51, the patient was diagnosed as having decreased luteal blood flow.

a; p < 0.01 and b; p < 0.05 v.s. control (Kruskal-Wallis test followed by the Mann-Whitney *U*-test using the Bonferroni correction). c; p < 0.01 v.s. control (x^2^-test with Bonferroni correction).

We further focused on the CL-RI of each corpus luteum in case of the patients with multiple ovulations (Figure 1). In patients with multiple corpora lutea, CL-RI did not vary much among the corpora lutea (Figure 1). The mean CL-RI of corpora lutea in the control group (0.552 ± 0.013) was more than the cutoff value; only 3 of the 17 corpora lutea in this group had CL-RI < 0.51 (Table 3). Treatments with HCG or vitamin E significantly improved the CL-RI to less than 0.51, and the number of corpora lutea with CL-RI < 0.51 was 18 out of 21 corpora lutea in the HCG group and 16 out of 18 corpora lutea in the vitamin E group (Table 3).

**Table 3:** Corpus luteum-related CL-RI in the treatment groups

	No. of CL	CL-RI	No. of CL with CL-RI < 0.51
Control	17	0.552 ± 0.013	3/17

HCG	21	0.457 ± 0.011^a^	18/21^b^

Vitamin E	18	0.448 ± 0.012^a^	16/18^b^

Corpus luteum-resistance index (CL-RI) was measured in each corpus luteum of the patient during the mid-luteal phase in the control group, HCG group, and vitamin E group. The table summarizes the data in Figure 1. The value shows the mean ± SEM from the CL-RI of each corpus luteum. In this study, when CL-RI was more than 0.51, the corpus luteum was evaluated as having decreased blood flow.

a; p < 0.01 v.s. control (Kruskal-Wallis test followed by the Mann-Whitney *U*-test using the Bonferroni correction). b; p < 0.01 v.s. control (x^2^-test with Bonferroni correction).

## Discussion

Our results show that patients undergoing the GnRHa long protocol have high blood flow impedance of the corpus luteum with very low serum LH levels, and that HCG treatment significantly improved blood flow impedance of the corpus luteum. Because high blood flow impedance of the corpus luteum in patients with luteal phase defect was improved by HCG administration [5], it is likely that LH is involved in the regulation of luteal blood flow.

Interestingly, in patients with multiple corpora lutea, CL-RI did not vary much among the individual corpora lutea, which suggests that CL-RI is influenced by endocrine factors.

Luteal phase defect has various causes. The GnRHa long protocol is known to cause luteal phase defect because it drastically suppresses serum LH levels. Luteal blood flow is closely related to luteal function [4,5]. The decrease in luteal blood flow is a critical factor in luteal phase defect [9-12]. Therefore, luteal phase defect caused by GnRHa long protocol is due not only to low serum LH levels but also to the decreased luteal blood flow.

The present study showed vitamin E has an ability to improve luteal blood flow impedance, in agreement with previous studies that showed vitamin E increases blood flow in a variety of organs including corpora lutea and endometrium [5,13-15].

Although HCG has an ability to improve luteal blood flow impedance, the mechanism is unclear. In the present study, HCG injection on days 2 and 4 after ovulation induction decreased luteal blood flow impedance. It is, therefore, likely that HCG influences luteal blood flow through some mediators rather than by its direct action [16]. One possible mediator is VEGF, which stimulates angiogenesis in the corpus luteum [17-19], and VEGF expression is increased by HCG [20-23]. HCG may, therefore, increase luteal blood flow by stimulating angiogenesis in the corpus luteum. HCG may also work through vasoactive substances such as nitric oxide (NO) or endothelin [24,25]. HCG increases NO synthase expression in the ovary of the rat and sheep [26,27], and increases rat ovarian blood flow via locally produced NO [28]. Endothelin-1, a vasoconstrictor, is produced by luteal cells [29], and HCG may affect luteal blood flow by regulating endothelin-1 [30]. However, further studies are needed to determine whether these factors have a role in the mechanism by which HCG increases luteal blood flow.

## Conclusions

The present results show that the GnRHa long protocol causes high blood flow impedance of the corpus luteum and very low serum LH levels. Our result also showed that HCG administration decreases luteal blood flow impedance. Taken together, these results strongly suggested that luteal blood flow is regulated by LH.

## Competing interests

The authors declare that they have no competing interests.

## Authors' contributions

AT conceived of the study, carried out the ultrasonographic studies, and performed the statistical analysis. IT, FK, RL, RM, HA, TT, HT, KS, and HM carried out the ultrasonographic studies. NS conceived of the study, and participated in its design and coordination. All authors read and approved the final manuscript.
